# Comparison of Effectiveness and Safety of Dual Antiplatelet Therapy (DAPT) With Clopidogrel and Aspirin Versus Aspirin Monotherapy in Patients With Mild-to-Moderate Stroke and Transient Ischemic Attack: A Systematic Review and Meta-Analysis

**DOI:** 10.7759/cureus.58909

**Published:** 2024-04-24

**Authors:** Tanya Sinha, Areeba Riaz, Anurag Rawat, Chaw N Phoo, Maymona E Nageye, Sandipkumar S Chaudhari, Calvin R Wei, Adil Amin

**Affiliations:** 1 Medical Education, Tribhuvan University, Kirtipur, NPL; 2 Medicine, Quiad-e-Azam Medical College, Bahawalpur, PAK; 3 Interventional Cardiology, Himalayan Institute of Medical Sciences, Dehradun, IND; 4 Internal Medicine, University of Medicine, Mandalay, Mandalay, MMR; 5 Internal Medicine - Pediatrics, Avalon University School of Medicine, Willemstad, CUW; 6 Cardiothoracic Surgery, University of Alabama at Birmingham, Birmingham, USA; 7 Family Medicine, University of North Dakota School of Medicine and Health Sciences, Fargo, USA; 8 Research and Development, Shing Huei Group, Taipei, TWN; 9 Cardiology, Pakistan Navy Ship Shifa, Karachi, PAK

**Keywords:** transient ischemic attack, stroke, aspirin, clopidogrel, dual anti-platelet therapy

## Abstract

The aim of this meta-analysis was to assess the effectiveness and safety of the combination of clopidogrel and aspirin in patients with mild ischemic stroke or transient ischemic attack (TIA). The methodologies employed in this meta-analysis strictly followed the commonly used reporting formats for systematic reviews and meta-analyses. The methodologies employed in this meta-analysis strictly followed the Preferred Reporting Items for Systematic Reviews and Meta-Analyses (PRISMA). Until March 25, 2024, we conducted thorough searches on PubMed, EMBASE (Excerpta Medica Database), and the Cochrane Library to locate studies investigating the efficacy and safety of dual antiplatelet therapy (DAPT) in patients with mild or moderate stroke or TIA. Outcomes assessed in this meta-analysis included stroke (including ischemic stroke and hemorrhagic stroke), myocardial infarction, all bleeding events, and moderate to severe bleeding events.

A total of 12 studies were included in this meta-analysis. The total number of enrolled patients across these studies was 35,369, with 16,957 receiving DAPT and 18,412 receiving aspirin monotherapy. The risk of developing stroke was significantly lower in patients receiving the combination of clopidogrel and aspirin compared to the aspirin monotherapy group (relative risk (RR): 0.77, 95% confidence interval (CI): 0.72 to 0.83, p-value<0.0001). No significant differences were there in terms of all bleeding events (RR: 1.37, 95% CI: 0.92 to 2.04, p-value: 0.12) and moderate to severe bleeding events (RR: 1.18, 95% CI: 0.86 to 1.63, p-value: 0.30). These findings highlight the importance of carefully weighing the potential benefits against the risks, especially in clinical decision-making for patients with TIA or ischemic stroke. Further research is warranted to elucidate optimal strategies for balancing stroke prevention with bleeding risk mitigation in this patient population.

## Introduction and background

Approximately 65% of all ischemic cerebrovascular events are constituted as acute mild ischemic stroke or transient ischemic attack (TIA) [1]. A recent comprehensive prospective study revealed an 8% risk of subsequent ischemic stroke within one week for mild stroke and high-risk TIA cases [2], with a 10.5% risk within three months following the initial event [3]. Both the Clopidogrel in High-Risk Patients with Acute Nondisabling Cerebrovascular Events (CHANCE) trial and the Platelet-Oriented Inhibition in New TIA and Minor Ischemic Stroke (POINT) trial demonstrated the effectiveness of dual antiplatelet therapy (DAPT) involving clopidogrel and aspirin in preventing recurrent cerebrovascular events among patients with mild stroke (National Institutes of Health Stroke Scale (NIHSS) score ≤3) or heightened risk of TIA within 24 hours of symptom onset. Therefore, the combination antiplatelet regimen serves as a crucial antithrombotic strategy against early vascular events [4-5].

Extensive documentation supports the effectiveness of antiplatelet therapy in preventing secondary strokes. Aspirin holds a central position in the treatment or prevention of non-cardioembolic strokes, including TIA and ischemic strokes. Previous trials have firmly established aspirin's efficacy, making it widely accepted as the standard antiplatelet therapy for patients with non-cardioembolic strokes [6]. While DAPT is increasingly used in ischemic heart disease, there is limited robust evidence supporting its effectiveness in preventing strokes. Nevertheless, a recent systematic review suggests that DAPT can safely and effectively reduce the recurrence of strokes and combined vascular events in patients with TIA or ischemic strokes when compared to monotherapy [7].

Clopidogrel, an adenosine-diphosphate (ADP)-receptor antagonist, demonstrates clinical advantages over aspirin in both ex-vivo platelet aggregation and thrombosis in animal models [8]. Combining clopidogrel with aspirin shortly after a minor ischemic stroke or TIA has been shown to mitigate the early risk of new stroke without elevating the risk of bleeding [4,9]. Previous meta-analyses have underscored the benefits of incorporating clopidogrel alongside aspirin in patients with stroke or TIA [10].

Recent studies have emerged to assess the efficacy of adding clopidogrel in this patient cohort. Hence, we have conducted an updated systematic review and meta-analysis encompassing patients with mild ischemic stroke or high-risk TIA within three days of presentation. This review aims to elucidate the effectiveness and safety of DAPT with clopidogrel and aspirin versus aspirin monotherapy for mild ischemic stroke or TIA.

## Review

Methodology

The methodologies employed in this meta-analysis strictly followed the Preferred Reporting Items for Systematic Reviews and Meta-Analyses (PRISMA) guidelines.

Literature Retrieval 

Until March 25, 2024, we conducted thorough searches on PubMed, EMBASE (Excerpta Medica Database), and the Cochrane Library to locate studies investigating the efficacy and safety of DAPT in patients with mild or moderate stroke or TIA. Our database searches utilized specific medical subject headings and keywords, including (DAPT or dual antiplatelet therapy or clopidogrel plus aspirin) AND (aspirin alone OR aspirin monotherapy). We did not impose any further restrictions on the relevant records identified through this method. Furthermore, we manually searched the reference lists of potentially eligible papers and previous systematic reviews to identify any additional studies that may have been missed during the initial database search.

Selection of Studies

The detailed inclusion criteria were as follows: (a) Patients with mild to moderate stroke or transient ischemic attack (TIA), (b) Administration of clopidogrel alongside aspirin as intervention, (c) aspirin monotherapy in the control group, and (d) stroke (including ischemic and hemorrhagic stroke), myocardial infarction, all bleeding events, and moderate-to-severe bleeding events as outcomes. Excluded from consideration were correspondences, proceedings, editorials, expert opinions, evaluations lacking primary data, and non-human studies. Additionally, duplicate publications were disregarded, and studies lacking a comparison or control group were also excluded.

Data Extraction

Two researchers independently gathered details from each study. Any discrepancies were resolved through discussion until a consensus was reached. The following information was collected from each eligible study: trial name, publication year, average age of patients, variables concerning the interventions, number of male patients, and outcomes of interest for each group.

Statistical Analysis

Data synthesis and analysis involved combining relative risks (RRs) and their corresponding 95% confidence intervals (CIs) to assess the safety and efficacy of DAPT compared to aspirin monotherapy. Heterogeneity across study trials was evaluated using I^2^ tests and Chi-squared tests to determine the appropriate analysis model (random-effects model or fixed-effects model). Studies with an I^2^ value ≥ 50% were considered to exhibit moderate to high heterogeneity, while those with an I^2^ value < 50% were considered to have low heterogeneity. For studies demonstrating low heterogeneity, the fixed-effects model was employed, whereas the random-effects model was used for pooled RR calculations. Statistical significance was defined as p < .05. The statistical analysis was performed utilizing Review Manager version 5.4.1 software (RevMan, The Cochrane Collaboration, Oxford, United Kingdom), and the outcomes of the meta-analysis were visualized through forest plots.

Results

**Figure 1:**
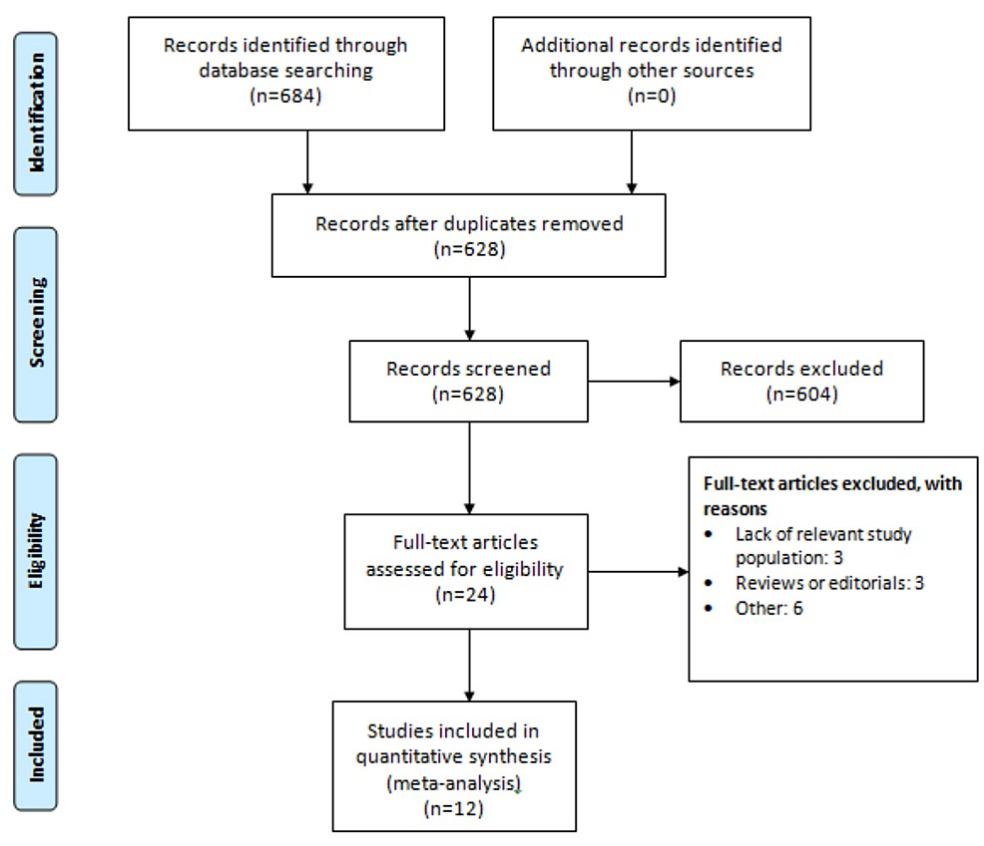
Preferred Reporting Items for Systematic Reviews and Meta-Analyses (PRISMA) flowchart of study selection

Among the 684 studies identified through the search strategy, 24 underwent a thorough examination of their full texts. Ultimately, 12 studies meeting the inclusion criteria were included in this meta-analysis. A depiction of the selection process is presented in Figure 1, while Table 1 provides an overview of the characteristics of the included trials. The total number of enrolled patients across these studies was 35,369, with 16,957 receiving DAPT and 18,412 receiving aspirin monotherapy.

**Table 1:** Characteristics of included studies RCT: Randomized-control trial; RC: Retrospective cohort; C+A: Clopidogrel+aspirin; A: Aspirin

Author	Year	Study Design	Region	Groups	Sample Size	Mean age (years)	Males (n)
Chen et al. [11]	2024	RCT	China	C+A	1502	65.7	972
				A alone	1413	66.1	923
Fan et al. [12]	2022	RC	China	C+A	375	60	271
				A alone	131	63	96
Geraghty et al. [13]	2010	RC	United Kingdom	C+A	247	71.2	127
				A alone	286	68.6	171
Hankey et al. [14]	2011	RCT	Multinational	C+A	2157	64.8	1350
				A alone	2163	64.9	1380
He et al. [15]	2015	RCT	China	C+A	321	62.9	183
				A alone	326	61.5	185
Johnston et al. [5]	2018	RCT	Multinational	C+A	2432	65	1335
				A alone	2449	65	1351
Kennedy et al. [16]	2007	RCT	Canada	C+A	198	67.1	61
				A alone	194	68.9	52
Lee et al. [17]	2020	RC	Korea	C+A	6760	66.9	4377
				A alone	8470	64.9	5194
Markus et al. [18]	2005	RCT	Multinational	C+A	51	66.4	35
				A alone	56	62.8	39
Wang et al. [4]	2013	RCT	Multinational	C+A	2584	63	1732
				A alone	2586	62	1688
Wong et al. [19]	2010	RCT	Multinational	C+A	46	59.2	36
				A alone	52	56.4	40
Yi et al. [20]	2014	RCT	China	C+A	284	69.2	156
				A alone	286	70.1	157

Meta-Analysis of Outcomes 

**Figure 2:**
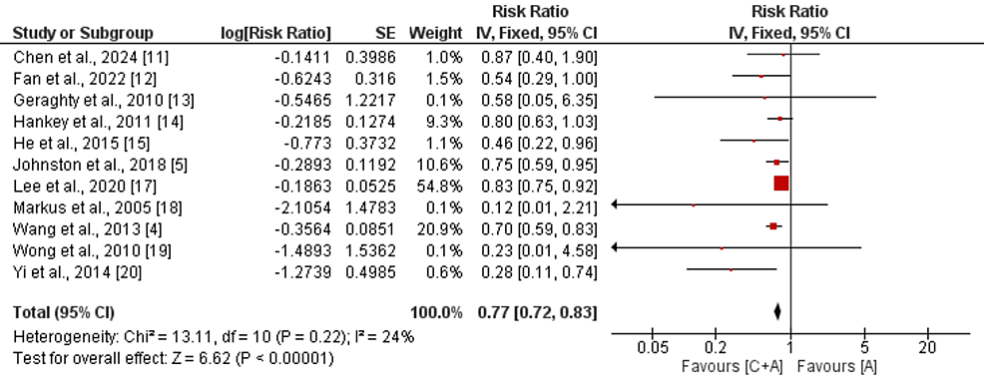
Comparison of risk of developing stroke C+A: Clopidogrel+aspirin; A: Aspirin; SE: Standard error; IV: Inverse of variance [4-5,11-15,17-20]

Stroke: Eleven studies compared the risk of developing stroke between patients who received the combination of clopidogrel and aspirin and patients who received aspirin alone. As shown in Figure 2, the risk of developing stroke was significantly lower in patients receiving the combination of clopidogrel and aspirin compared to the aspirin monotherapy group (RR: 0.77, 95% CI: 0.72 to 0.83, p-value<0.0001). No significant heterogeneity was reported among the study results (I^2^: 24%). 

All Bleeding Events 

**Figure 3:**
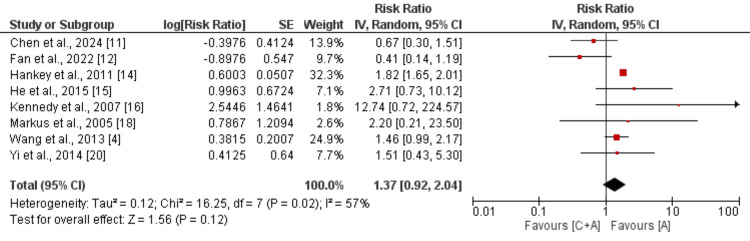
Comparison of risk of all bleeding events C+A: Clopidogrel+aspirin; A: Aspirin; SE: Standard error; IV: Inverse of variance [4,11-12,14-16,18,20]

Eight studies were included in the pooled analysis of comparing the effect of clopidogrel and aspirin and aspirin monotherapy on decreasing the risk of all bleeding events in patients with mild to moderate stroke or TIA and the results of the pooled analysis are shown in Figure 3. Pooled analysis showed that the risk of all bleeding events was lower in patients receiving aspirin alone. However, the difference was statistically insignificant (RR: 1.37, 95% CI: 0.92 to 2.04, p-value: 0.12). Significant heterogeneity was reported among the study results (I^2^: 57%).

*Moderate-to-Severe Bleeding Events* 

**Figure 4:**
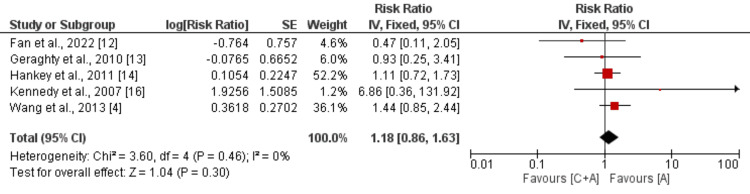
Comparison of risk of moderate to severe bleeding events C+A: Clopidogrel + aspirin; A: Aspirin; SE: Standard error; IV: Inverse of variance [4,12-14,16]

Five studies were incorporated into the combined analysis, comparing the risk of moderate to severe bleeding events among patients receiving clopidogrel and aspirin versus aspirin monotherapy in individuals with mild to moderate stroke or TIA. Illustrated in Figure 4, the risk of moderate to severe bleeding events did not exhibit significant differences between the two groups (RR: 1.18, 95% CI: 0.86 to 1.63, p-value: 0.30). Notably, no significant heterogeneity was observed among the study results (I^2^: 0%).

*Myocardial Infarction* 

**Figure 5:**
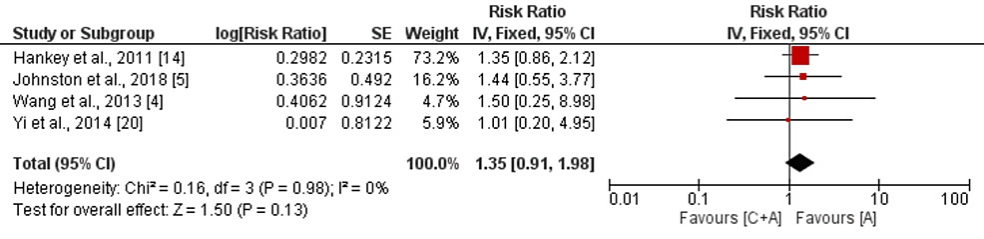
Comparison of risk of myocardial infarction C+A: Clopidogrel + aspirin; A: Aspirin; SE: Standard error; IV: Inverse of variance [4-5,14,20]

Four studies were incorporated into the combined analysis, comparing the impact of clopidogrel and aspirin versus aspirin monotherapy in reducing the risk of myocardial infarction among patients with mild to moderate stroke or TIA. The pooled analysis results are illustrated in Figure 5. The pooled analysis indicated that there was no statistically significant difference in the risk of myocardial infarction between the two groups (RR: 1.35, 95% CI: 0.91 to 1.98, p-value: 0.13). It's noteworthy that there was no significant heterogeneity observed among the study findings (I^2^: 0%). 

Discussion

The meta-analysis comprised 12 studies that assessed the effectiveness and safety of clopidogrel combined with aspirin versus aspirin monotherapy in patients with mild to moderate stroke and TIA. Results indicated a significantly lower risk of stroke development in patients receiving the combination therapy of aspirin and clopidogrel compared to those on aspirin alone. However, the pooled analysis revealed a higher risk of bleeding events among patients receiving combination therapy compared to aspirin monotherapy, although this disparity did not reach statistical significance. In a meta-analysis conducted by Ye et al. [10], the addition of clopidogrel to aspirin for patients with TIA or ischemic stroke was associated with a notable reduction in the risk of ischemic stroke recurrence, albeit with a potential increase in bleeding risk compared to aspirin monotherapy.

The combined use of aspirin and clopidogrel has been shown to synergistically inhibit platelet aggregation [21]. This combination therapy has demonstrated efficacy in reducing the recurrent ischemic events risk in individuals with acute coronary syndrome (ACS) [8,22]. Results from the Clopidogrel and Aspirin for Reduction of Emboli in Symptomatic Carotid Stenosis (CARESS) study revealed that the combining of clopidogrel with aspirin resulted in a decrease in large-vessel atherosclerotic stroke [18]. Furthermore, the Fast Assessment of Stroke and TIA to Prevent Early Recurrence (FASTER) trial [16] demonstrated a significant decrease in early recurrent stroke risk when aspirin was combined with clopidogrel compared to aspirin alone.

There were various possible reasons for the observed decrease in stroke occurrences within the clopidogrel-aspirin cohort. Yi et al.'s study [20] revealed that patients who later experienced neurological deterioration or recurrent stroke exhibited higher levels of arachidonic acid (AA)-induced or adenosine diphosphate (ADP)-induced platelet aggregation and platelet-leukocyte aggregates upon initial admission, compared to those who did not encounter such issues. These results indicate a possible connection between platelet aggregation and platelet-leukocyte aggregates in the development of recurrent stroke or neurological deterioration subsequent to acute large-artery atherosclerosis stroke. Moreover, it seems that the quantity instead of the specific kind of antiplatelet drugs plays a more significant role in reducing stroke recurrence. Different combinations of antiplatelet agents demonstrate synergistic effects based on distinct antiplatelet mechanisms. As a result, combined antiplatelet therapy may reduce the incidence of recurrent ischemic stroke or neurological deterioration. On the contrary, during the initial phase of TIA or ischemic stroke, combining two antiplatelet therapies diminishes the occurrence of microembolic signals in patients predominantly diagnosed with intracranial symptomatic stenosis, as opposed to using a single therapy alone [23].

While the combination of clopidogrel and aspirin has demonstrated a favorable effect in reducing the risk of recurrent stroke events, it is accompanied by an increased risk of bleeding events. The risk of bleeding remains a primary safety consideration when administering the combination treatment of clopidogrel and aspirin in clinical practice. Maintaining a delicate balance between reducing stroke recurrence and managing the elevated risk of major bleeding events is crucial.

Concerns arise due to several factors, including older age, prior use of antiplatelet agents, and stroke presentation as a qualifying event (in comparison to TIA) [24]. Additionally, the antiplatelet therapy duration could be a vital indicator of an increased bleeding risk with DAPT. Findings from the Management of Atherothrombosis with Clopidogrel in High-risk Patients (MATCH) trial indicated that bleeding complications associated with clopidogrel plus aspirin remained consistent over time, highlighting a temporal window (approximately three months) during which the risk of bleeding outweighed the benefits in high-risk patients with ischemic stroke or TIA [25]. Furthermore, the utilization of a loading dose has been observed to result in a more rapid inhibition of platelet aggregation, leading to an antithrombotic effect evident within 90 minutes and peaking within six hours [26]. Moreover, there's a suggestion that higher doses of aspirin may be less effective than lower doses in preventing vascular events [27].

Study Limitations

In this systematic analysis, it is important to acknowledge several limitations. Firstly, the cumulative influence of multiple factors was apparent, including limitations related to conducting a study-level meta-analysis due to the absence of patient-level data. It's crucial to account for clinical heterogeneity among the studies when interpreting our findings. Secondly, subgroup analysis of significant risk indicators in the two cohorts was limited because high-risk patients may be more susceptible to complications, thus restricting the availability of relevant subgroup data in the literature. Consequently, analyses concerning important risk indicators, such as the use of a loading dose and various stroke subtypes, were not performed in this analysis. Future studies are necessary to clarify the impact of different variables on the effectiveness and safety of DAPT.

## Conclusions

In conclusion, our meta-analysis of 12 studies, encompassing a substantial cohort of 18,469 patients with TIA or ischemic stroke, underscores the efficacy of clopidogrel-aspirin combination therapy in reducing the risk of stroke. However, despite its benefits in stroke prevention, no significant difference in bleeding risk was identified when compared to aspirin monotherapy. These findings highlight the importance of carefully weighing the potential benefits against the risks, especially in clinical decision-making for patients with TIA or ischemic stroke. Further research is warranted to elucidate optimal strategies for balancing stroke prevention with bleeding risk mitigation in this patient population.
